# SMARCB1 (INI1)-deficient malignant melanocytic neoplasm with histologically confirmed vertebral metastases in a young adult: a case report

**DOI:** 10.3389/fonc.2026.1845400

**Published:** 2026-07-15

**Authors:** Ibrahim Al-Hassana, Lethongsavarn Vincent, Setcheou Alihonou, Sieleche Christian, Mahamadou Ango Souleymane, Rabhi Mohammed, Do Laurent

**Affiliations:** Department of Neurosurgery, Centre Hospitalier Universitaire Pointe-a-Pitre Abymes, Pointe-a-Pitre, Guadeloupe

**Keywords:** INI1-deficient tumor, melanocytic differentiation, rhabdoid Tumor, SMARCB1, uveal neoplasm, vertebral metastasis

## Abstract

**Background:**

SMARCB1 (INI1)-deficient tumors are rare aggressive neoplasms characterized by biallelic inactivation of the SMARCB1 gene and loss of nuclear INI1 protein expression. Although melanocytic differentiation has occasionally been reported in SMARCB1-deficient tumors, intraocular presentations with histologically confirmed vertebral metastases in adults remain exceptionally uncommon and diagnostically challenging.

**Case presentation:**

A 21-year-old woman presented with progressive left retro-orbital pain and visual impairment. Ophthalmologic imaging revealed a choroidal lesion initially suspected to represent uveal melanoma. Enucleation of the left eye demonstrated a highly cellular malignant epithelioid neoplasm arising in the choroid with focal spindle-cell areas, rhabdoid features, marked cytologic atypia, and focal melanocytic differentiation. Immunohistochemistry showed diffuse positivity for S100 and Melan-A, focal HMB45 expression, retained BAP1 expression, and complete loss of INI1 expression. Molecular analysis identified a pathogenic truncating SMARCB1 alteration, c.656C>G, p.(Ser219*), associated with homozygous deletion of the SMARCB1 locus. Additional testing revealed no pathogenic alterations involving canonical uveal melanoma-associated genes, including GNAQ, GNA11, CYSLTR2, SF3B1, EIF1AX, and BAP1. One year after diagnosis, the patient developed multifocal painful vertebral lesions. Histopathological examination of a T8 vertebral biopsy revealed a poorly differentiated malignant neoplasm with extensive necrosis and rhabdoid morphology. Tumor cells demonstrated diffuse S100 positivity, focal HMB45 expression, complete loss of INI1, and absence of epithelial or myogenic differentiation (negative cytokeratins, desmin, and myogenin). Expert pathology review supported metastatic dissemination of a SMARCB1-deficient malignant neoplasm with melanocytic differentiation. The patient received palliative radiotherapy (30 Gy in 10 fractions to D7–D9) followed by systemic chemotherapy with etoposide and ifosfamide. Despite multimodal treatment, progressive disease occurred and the patient died three years after the initial diagnosis.

**Conclusions:**

This case expands the clinicopathologic spectrum of SMARCB1-deficient tumors and highlights the diagnostic complexity of neoplasms exhibiting melanocytic differentiation. Integrated histopathologic, immunophenotypic, and molecular assessment is essential to avoid diagnostic misclassification, particularly in unusual intraocular presentations.

## Introduction

SMARCB1 (INI1)-deficient tumors constitute a heterogeneous group of aggressive neoplasms characterized by biallelic inactivation of the SMARCB1 tumor suppressor gene and complete loss of nuclear INI1 protein expression (1–3). These tumors encompass atypical teratoid/rhabdoid tumors (AT/RT), malignant rhabdoid tumors, epithelioid sarcoma, and a growing spectrum of extra-central nervous system malignancies exhibiting divergent differentiation patterns (2–4). Loss of SMARCB1 disrupts the SWI/SNF chromatin remodeling complex, resulting in epigenetic dysregulation and tumor progression (3).

Melanocytic differentiation in SMARCB1-deficient tumors remains exceptionally rare. Conventional uveal melanoma, the most common primary intraocular malignancy in adults, typically retains INI1 expression and is molecularly characterized by activating alterations involving GNAQ, GNA11, CYSLTR2, SF3B1, EIF1AX, or BAP1 (5, 6). Recently, Cyrta et al. described two cases of SMARCB1-deficient malignant melanocytic uveal tumors, suggesting the existence of a distinct neural crest-derived tumor entity associated with SMARCB1 alterations. Given the rarity of these neoplasms, additional well-documented observations are required to better define their clinicopathologic spectrum, metastatic potential, and clinical outcome.

The present report describes an additional case of SMARCB1-deficient malignant melanocytic choroidal tumor with histologically confirmed vertebral metastases and compares its clinical and pathological features with those previously reported in the literature.

## Case presentation

### Patient information and clinical presentation

A 21-year-old woman presented with progressive left retro-orbital pain associated with progressive visual impairment. No significant past medical history or relevant familial oncologic history was reported. Ophthalmologic imaging demonstrated a choroidal mass involving the posterior segment of the left eye, initially interpreted as highly suggestive of uveal melanoma. Owing to persistent symptoms and concern for malignancy, left ocular enucleation was performed.

### Pathological examination of the primary ocular tumor

#### Gross findings

The enucleated globe measured 28 × 28 × 23 mm. The optic nerve measured 4 mm in diameter and 3 mm in length. On sectioning, a burgundy-to-whitish-gray choroidal mass measuring 35 × 28 × 4 mm was identified within the posterior and lateral choroid, extending toward the optic nerve head. Associated retinal detachment was present overlying the lesion. Focal scleral thinning was observed without gross extrascleral extension.

#### Histopathological findings

Microscopically, the tumor was centered in the choroid and consisted of a highly cellular proliferation arranged in nests and sheets of predominantly epithelioid cells with focal spindle-cell areas embedded within a fibrous stroma. Tumor cells displayed abundant eosinophilic cytoplasm, occasionally containing pigment, enlarged hyperchromatic nuclei, prominent eosinophilic nucleoli, and marked cytologic atypia. Focal rhabdoid features were identified.

Mitotic activity was elevated, reaching approximately 10 mitoses per 10 high-power fields. Tumor necrosis represented less than 10% of the tumor volume. Focal scleral invasion, including vascular invasion, was observed without extrascleral extension. Tumor infiltration involved the optic nerve up to the level of the lamina cribrosa without meningeal extension, and the posterior surgical margin remained free of tumor.

### Immunohistochemistry

Immunohistochemical analysis demonstrated strong diffuse positivity for S100 and Melan-A, with focal positivity for HMB45. Nuclear BAP1 expression was retained in all tumor cells. Complete loss of nuclear INI1 expression was observed in tumor cells, while internal non-neoplastic elements showed preserved expression.

Additional immunostains including SOX10, EMA, CD34, and PRAME could not be performed because of limited remaining material and represent a limitation of the present study.

### Molecular analysis

All molecular analyses were performed on the primary ocular tumor.

Genomic DNA extraction from formalin-fixed paraffin-embedded tissues was performed using the MACHEREY–NAGEL extraction system. Target enrichment was carried out using the SureSelect XT hs kit (Agilent Technologies) with a custom-designed DRAGON gene panel. Sequencing was performed on Illumina NextSeq or NovaSeq platforms according to manufacturer recommendations.

Raw sequencing data were processed using bcl2fastq, aligned to the human reference genome (GRCh37/hg19) using BWA-MEM, and analyzed through an adapted FACETS pipeline for copy number alterations. Variant calling was performed using VarScan2, and variants were annotated using Alamut and COSMIC databases.

Molecular testing identified a pathogenic truncating SMARCB1 variant, c.656C>G, p.(Ser219*), associated with homozygous deletion of the SMARCB1 gene on copy number variation analysis. Additional targeted analysis of genes frequently altered in uveal melanoma (GNAQ, GNA11, CYSLTR2, SF3B1, EIF1AX, and BAP1) revealed no pathogenic alterations.

Constitutional analysis additionally demonstrated deletion involving all nine exons of the **SMARCB1** gene.

DNA methylation profiling was not performed and represents a diagnostic limitation of this study.

### Metastatic vertebral disease

One year after initial diagnosis, the patient developed progressive back pain. Imaging revealed multifocal osteolytic vertebral lesions involving the thoracic spine (**Figures 1**–**3**).

**Figure 1:**
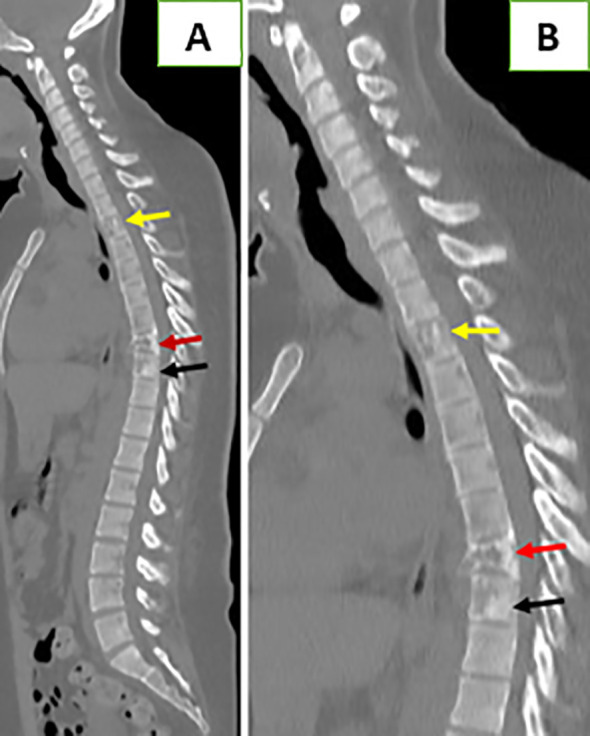
Sagittal CT scan, bone window. **(A)** Whole spine. **(B)** Cervicothoracic spine. Osteolysis without compression of the T3 vertebral body (yellow arrow). Osteolysis with wedge compression fracture of the T8 vertebral body (orange arrow). Osteolysis without compression of the T9 vertebral body (black arrow).

**Figure 2:**
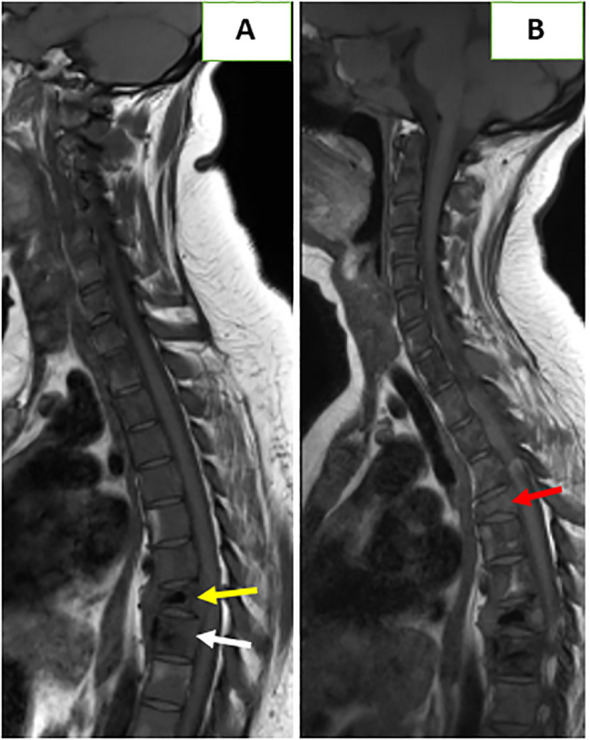
T1-weighted MRI of the cervicothoracic spine showing diffuse bone marrow infiltration with low T1 signal. Cemented compression fracture of T8 [yellow arrow, **(A)**]. Cemented non-compression fracture of T9 [white arrow, **(A)**]. Development of a compression fracture of T5 after 6 months of progression [orange arrow, **(B)**].

**Figure 3:**
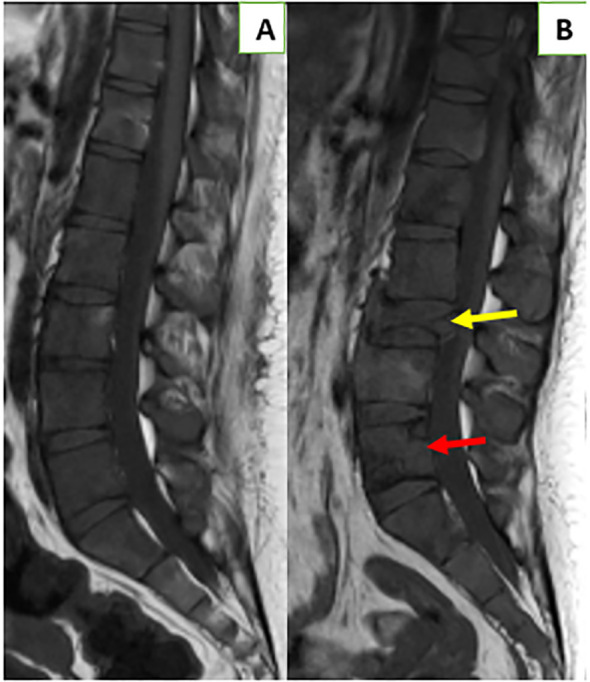
T1-weighted MRI of the lumbar spine showing diffuse bone marrow infiltration with low T1 signal. **(A)** No fracture or compression deformity. **(B)** Compression fracture of L3 (yellow arrow) and a non-compression fracture of L5.

Histopathological examination of a T8 vertebral biopsy demonstrated bone infiltrated by a poorly differentiated malignant neoplasm with extensive necrosis. Tumor cells were discohesive and medium-to-large in size with eosinophilic cytoplasm and eccentrically positioned nuclei. Although evaluation was partially limited by crush artifact, focal rhabdoid morphology was evident.

Immunohistochemical analysis demonstrated complete loss of INI1 expression with preserved staining in internal controls. Tumor cells exhibited diffuse strong S100 positivity and focal HMB45 positivity. Cytokeratins (AE1/AE3), desmin, and myogenin were negative, excluding epithelial and myogenic differentiation.

The mixed immunohistochemical profile demonstrates focal expression of the melanocytic markers Melan-A and HMB45, suggesting a tumor phenotype consistent with a neoplasm with melanocytic phenotype. This is associated with loss of INI1 expression, a characteristic feature of SMARCB1-deficient tumors. The absence of AE1/AE3 expression confirms that the lesion does not represent a carcinomatous process. Furthermore, the lack of desmin and myogenin expression excludes a malignant tumor of myogenic origin, despite the presence of cells with rhabdoid morphology.

The striking morphologic, immunophenotypic, and molecular concordance between the primary ocular tumor and vertebral lesion strongly supported metastatic dissemination from the original neoplasm. Diagnostic findings were independently reviewed and confirmed at a national expert pathology referral center (**Figure 4**).

**Figure 4:**
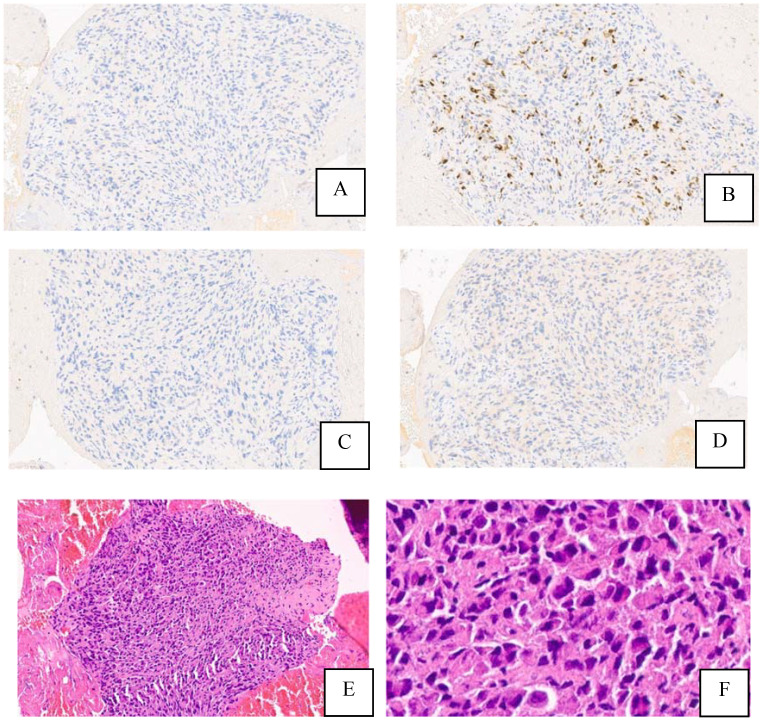
Histopathological and Immunohistochemical examination **(A)** Immunohistochemical staining for Desmin (×10) showing absence of cytoplasmic expression in tumor cells, not supporting myogenic differentiation **(B)** Immunostaining for MIB1 (x10) showing a high proliferation index around 30%, reflecting numerous cycling cells, indicative of tumor aggressiveness **(C)** Immunostaining for Myogenin (x10) showing no expression by neoplastic cells, not supporting skeletal (striated) muscle differentiation **(D)** Immunostaining for AE1/3 (x10) showing no expression by neoplastic cells, making it possible to rule out a carcinomatous process **(E, F)** Hematoxylin and eosin staining [**(E)** x9 and **(F)** x20] showing dissociated sheets of rhabdoid cells characterized by an abundant bright eosinophil cytoplasm with eccentrically located nuclei. The nuclei show atypia, with dense chromatin, irregular contours, and marked anisokaryosis.

### Therapeutic intervention and outcome

The patient underwent multimodal treatment including surgery (**Figure 5**), palliative radiotherapy (30 Gy delivered in 10 fractions to vertebral levels D7–D9), and systemic chemotherapy combining etoposide and ifosfamide. Despite treatment, disease progression occurred with worsening metastatic burden. The patient died three years after the initial diagnosis (**Table 1**).

**Figure 5:**
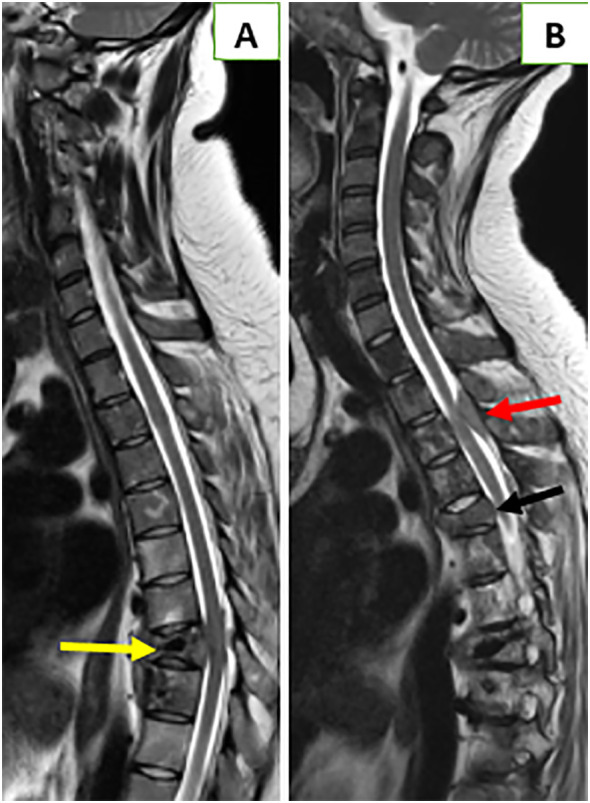
T2-weighted MRI of the lumbar spine showing a compression fracture of T8 treated with cement [**(A)**, yellow arrow]. After 6 months of progression, appearance of a compression fracture of T5 without posterior wall retropulsion [**(B)**, black arrow], and development of posterior epiduritis at the level of T3 [**(B)**, orange arrow].

**Table 1:** Clinical Timeline and Key Interventions.

Time point	Clinical event	Imaging	Intervention	Outcome
Initial	Retro-orbital pain, visual loss	MRI/US: choroidal mass	Corticosteroids	Temporary symptom relief
Month 1	Persistent symptoms	Biopsy	Left enucleation	Pathology: hybrid INI1-deficient tumor
Month 12	Bone pain	CT/MRI: T8, L3, ischium lesions	Biopsy, vertebroplasty, radiotherapy	Temporary pain relief
Month 18	Neurologic decline	MRI: cord compression	Decompression T2–T5	Partial neurologic improvement
Month 36	Progressive disease	MRI/CT: multi-level metastases	Supportive care	Death

## Discussion

This case illustrates a diagnostically challenging SMARCB1-deficient malignant neoplasm with melanocytic differentiation presenting in an unusual intraocular location and subsequently metastasizing to the vertebral column.

The differential diagnosis initially included conventional uveal melanoma. However, several features argued against this interpretation. Although the tumor demonstrated melanocytic differentiation with positivity for S100, Melan-A, and HMB45, conventional uveal melanomas typically retain INI1 expression and are molecularly characterized by activating alterations involving GNAQ, GNA11, CYSLTR2, EIF1AX, SF3B1, or BAP1 (5, 6). In the present case, retained BAP1 expression, complete INI1 loss, pathogenic truncating SMARCB1 alteration, homozygous deletion of SMARCB1, and absence of canonical uveal melanoma-associated alterations strongly argued against a conventional melanocytic malignancy.

The morphologic and molecular profile instead overlapped with the recently described SMARCB1-deficient malignant melanocytic uveal tumors reported by Cyrta et al. (7). Similar to those cases, our tumor demonstrated melanocytic marker expression, rhabdoid morphology, complete loss of INI1 expression, and absence of classical uveal melanoma-associated molecular alterations. However, our observation differs by the occurrence of histologically confirmed multifocal vertebral metastases, emphasizing the aggressive metastatic potential of this emerging clinicopathologic entity.

To our knowledge, only two comparable cases of SMARCB1-deficient malignant melanocytic uveal tumors have been reported to date by Cyrta et al. The clinicopathologic characteristics of these cases and the present observation are summarized in **Table 2**.

**Table 2:** Clinicopathologic comparison of reported SMARCB1-deficient melanocytic uveal tumors and the present case.

Study	Age/sex	Primary site	Histopathology	Immunophenotype	SMARCB1 status	Canonical uveal melanoma alterations	Metastatic pattern	Outcome
Cyrta et al., 2025 (7) (Patient 1)	23/F	Choroid	Poorly differentiated malignant melanocytic tumor with rhabdoid features	Melan-A+, HMB45+, SOX10+, diffuse INI1 loss	Biallelic SMARCB1 inactivation; germline alteration identified	Absent	Early bone metastases	Aggressive course
Cyrta et al., 2025 (7) (Patient 2)	14/F	Choroid	Epithelioid malignant melanocytic tumor with rhabdoid morphology	Melan-A+, HMB45+, SOX10+, diffuse INI1 loss	Biallelic SMARCB1 inactivation; germline alteration identified	Absent	No distant metastasis at report	Alive at follow-up
Present case	21/F	Choroid	Highly cellular epithelioid neoplasm with focal spindle-cell component, rhabdoid features, marked atypia, focal pigmentation	S100+, Melan-A+, focal HMB45+, retained BAP1, complete INI1 loss; SOX10 not performed	*Pathogenic SMARCB1 truncating mutation c.656C>G p.(Ser219) associated with homozygous deletion; constitutional deletion involving all 9 exons*	No pathogenic alterations in GNAQ, GNA11, CYSLTR2, SF3B1, EIF1AX, or BAP1	Histologically confirmed multifocal vertebral metastases	Death 3 years after diagnosis

INI1, integrase interactor 1; SMARCB1, SWI/SNF-related matrix-associated actin-dependent regulator of chromatin subfamily B member 1.

**Table 2** summarizes all currently reported SMARCB1-deficient melanocytic uveal tumors available in the literature and highlights the distinctive metastatic pattern and survival outcome observed in the present case.

All reported patients were young females presenting with primary choroidal tumors showing melanocytic differentiation, rhabdoid morphology, complete loss of INI1 expression, and absence of canonical uveal melanoma-associated molecular alterations. These shared features support the existence of an emerging clinicopathologic entity within the spectrum of SMARCB1-deficient melanocytic neoplasms.

However, clinically relevant differences were observed. One patient reported by Cyrta et al. developed early bone metastases and experienced an aggressive disease course, whereas the second patient remained disease-free at the time of publication. In comparison, our patient developed histologically confirmed multifocal vertebral metastases approximately 12 months after diagnosis and died 36 months after the initial presentation despite multimodal treatment including surgery, radiotherapy, and chemotherapy.

This comparison further expands the currently limited knowledge regarding the biological behavior of SMARCB1-deficient melanocytic uveal tumors and highlights their potential for aggressive metastatic dissemination.

SMARCB1-deficient malignant epithelioid neoplasms, epithelioid schwannoma-like tumors, and malignant peripheral nerve sheath tumors with melanocytic differentiation were also considered in the differential diagnosis (4, 8). However, the absence of convincing Schwannian morphology and the integrated immunophenotypic and molecular findings supported classification within the spectrum of SMARCB1-deficient malignant melanocytic neoplasms, rather than a conventional Schwannian or melanocytic tumor.

Accurate diagnosis of such rare neoplasms requires integrated assessment combining histopathology, immunophenotyping, and molecular profiling (7, 9). DNA methylation profiling, which may have provided additional diagnostic refinement, was not available in the present case and represents an important limitation.

Therapeutic management remains poorly defined because of the rarity of these tumors. Multimodal strategies combining surgery, radiotherapy, and chemotherapy are commonly employed, although prognosis generally remains poor in metastatic disease.

## Conclusion

We report an unusual case of a SMARCB1 (INI1)-deficient malignant neoplasm with melanocytic differentiation arising in the choroid of a young adult woman and subsequently metastasizing to the vertebral column. This case emphasizes the diagnostic complexity of neoplasms exhibiting overlapping melanocytic and rhabdoid features and underscores the critical role of integrated morphologic, immunophenotypic, and molecular evaluation in avoiding diagnostic misclassification.

## Data Availability

The raw data supporting the conclusions of this article will be made available by the authors, without undue reservation.
